# Refractive Outcome in Preterm Newborns With ROP After Propranolol Treatment. A Retrospective Observational Cohort Study

**DOI:** 10.3389/fped.2019.00479

**Published:** 2019-11-13

**Authors:** Luca Filippi, Giacomo Cavallaro, Lavinia Perciasepe, Elena Sandini, Gabriella Araimo, Giulia Regiroli, Genny Raffaeli, Paola Bagnoli, Massimo Dal Monte, Maura Calvani, Pina Fortunato, Silvia Osnaghi, Salvatore De Masi, Fabio Mosca

**Affiliations:** ^1^Neonatal Intensive Care Unit, Medical Surgical Fetal-Neonatal Department, “A. Meyer” University Children's Hospital, Florence, Italy; ^2^Neonatal Intensive Care Unit, Fondazione IRCCS Ca' Granda Ospedale Maggiore Policlinico, Milan, Italy; ^3^Department of Clinical Sciences and Community Health, University of Milan, Milan, Italy; ^4^Unit of General Physiology, Department of Biology, University of Pisa, Pisa, Italy; ^5^Oncohematology Unit, Department of Pediatric Oncology, “A. Meyer” University Children's Hospital, Florence, Italy; ^6^Pediatric Ophthalmology, A. Meyer” University Children's Hospital, Florence, Italy; ^7^Department of Ophthalmology, Fondazione IRCCS Ca' Granda, Ospedale Maggiore Policlinico, Università degli Studi di Milano, Milan, Italy; ^8^Clinical Trial Office, “A. Meyer” University Children's Hospital, Florence, Italy

**Keywords:** propranolol, beta-blocker, proliferative retinopathy, angiogenesis, preterm newborn

## Abstract

**Background:** Recent explorative studies suggest that propranolol reduces retinopathy of prematurity (ROP) progression, but the short-term effects of propranolol treatment at 1 year of corrected age have not been extensively evaluated.

**Methods:** A multi-center retrospective observational cohort study was conducted to assess the physical development and the refractive outcome of infants with prior ROP treated with propranolol. Forty-nine infants treated with propranolol were compared with an equal number of patients who did not receive any propranolol therapy and represent the control group, with comparable anthropometrical characteristics and stages of ROP.

**Results:** The weight, length, and head circumference at 1 year of corrected age were similar between infants who had been treated, or not, with propranolol, without any statistically significant differences. Refractive evaluation at 1 year showed spherical equivalent values decreasing with the progression of ROP toward more severe stages of the disease, together with an increasing number of infants with severe myopia. On the contrary, no differences were observed between infants who had been treated with propranolol and those who had not.

**Conclusion:** This study confirms that the progression of ROP induces an increase of refractive errors and suggests that propranolol itself does not affect the refractive outcome. Therefore, if the efficacy of propranolol in counteracting ROP progression is confirmed by further clinical trials, the conclusion will be that propranolol might indirectly improve the visual outcome, reducing the progression of ROP.

## Introduction

Despite continuous progress in the clinical management of prematurely born newborns, Retinopathy of prematurity (ROP) remains an important cause of potentially avoidable visual impairment and blindness in children (1, 2). Prematurity, low birth weight and premature oxygen exposition are the main risk factors associated with ROP (3). While other factors such as sepsis, intraventricular hemorrhage, and necrotizing enterocolitis may play a role in ROP development (3), the duration and the entity of oxygen exposition represents the main factor involved in its development (4).

Even though the pathogenesis of ROP has not been completely clarified yet, it appears as an oxygen-dependent biphasic disease, where two specular phases are clearly detectable (5). The first phase follows the oxygen extrauterine exposure and is characterized by the down-regulation of retinal pro-angiogenic factors, such as Vascular Endothelial Growth Factor (VEGF) and Insulin-like Growth Factor 1 (IGF-1). This event induces growth arrest, or even regression, of the normal retinal vasculature (“avascular” or “ischemic” phase). However, this phase promotes the transition to the following hypoxic phase. In fact, the increased metabolic demands of the maturing retina conflict with the rudimentary vascularisation, inducing progressive hypoxia that, in turn, causes a localized up-regulation of proangiogenic factors. This second phase of ROP, apparently specular to the previous phase, is characterized by a diffuse vaso-proliferation and retinal neovascularization, and is therefore usually called “proliferative.” These two phases are strictly related to each other, because the entity of the vascular obliteration during the first ischemic phase affects the degree and severity of retinal hypoxia in the following phase, inducing the up-regulation of retinal VEGF and consequently the neovascularization. These new vessels can grow out from the retina into the vitreous, inducing retinal edema and hemorrhage because of the increased permeability of these abnormal new vessels. This process can induce the formation of an abnormal fibrovascular tissue that can produce traction on the retina, which may lead to retinal distortion or detachment (5, 6).

Beyond the risk of retinal detachment, infants with prior ROP are at increased risk of developing visual impairment, related to refractive errors such as severe myopia and strabismus (7, 8). For this reason, the detection of effective therapeutic strategies is a question of global interest.

The ablation of the peripheral retina with laser photocoagulation is the most commonly used treatment and has been considered as the gold standard of ROP treatment: laser burns destroy the full thickness of the peripheral retina, which prevents neovascularization, reducing the progression of the disease. However, the visual outcome after treatment is still poor, especially for zone I ROP (9). Additionally, laser treatment can induce some adverse effects such as loss of the visual field, high myopia, corneal edema, intraocular hemorrhage, cataract formation, intraocular pressure changes (10). An alternative treatment currently available is represented by the intravitreal injection of neutralizing anti-VEGF antibodies. This treatment avoids retinal destruction but is not free from complications and systemic or local adverse events. Moreover, neither short- nor long-term data regarding their safety profile are currently available. Therefore, further studies are needed to evaluate their effects on functional and neurodevelopmental outcomes (11). A recent meta-analysis suggests that laser photocoagulation is probably more efficient than VEGF antagonists (less retreatment incidence) but induces more eye complications and increased myopia (12), even though this aspect is still controversial among specialists (13). Therefore, there is an urgent need for new pharmacological approaches in the prevention and treatment of ROP.

In the last few years, propranolol, a non-selective β1- and β2-adrenoreceptor (β-AR) blocker, has become an emerging option for treatment of ROP. Propranolol is currently considered as the first-line therapy of choice for the treatment of infantile hemangiomas (14), thanks to its ability to suppress the production of pro-angiogenic factors (15, 16). Considering the numerous pathogenetic analogies between infantile hemangiomas and ROP (17), pre-clinical (18–21), and clinical studies have explored the safety and efficacy of propranolol in reducing ROP progression. Oral propranolol resulted in being effective in limiting ROP progression (22–25); however, it was not considered safe enough to recommend for use in preterm infants due to the high incidence of life-threatening events observed in newborns taking 1 or 2 mg/kg/day of propranolol (22). This observation suggested to explore the feasibility of a topical approach to ensure an appropriate retinal propranolol delivery. A preliminary study performed in mice with oxygen-induced retinopathy (OIR), a well-established model of ROP, demonstrated that topical propranolol had an efficacy comparable to that of subcutaneous or oral propranolol (26). Subsequently, in rabbits, we demonstrated that the administration of one drop of 25 μL of 0.1% propranolol applied to both eyes every 6 h for 5 consecutive days produced retinal concentrations similar to, but plasma concentrations significantly lower than those measured after 1 mg/kg/day oral administration (27). These studies suggested that topical eye application can constitute an alternative delivery route to systemic administration also in newborns, in order to avoid the risk of associated side effects. A first clinical trial performed in newborns with stage 2 ROP demonstrated that propranolol 0.1% eye micro-drops were well-tolerated, but not sufficiently effective (28). On the contrary, a second clinical trial demonstrated that propranolol eye micro-drops administered a higher concentration (0.2%) and started at an earlier stage of the disease (stage 1 ROP) reduced ROP progression and showed an excellent safety profile (29).

However, the short-term effects of propranolol treatment at 1 year of corrected age have not been extensively evaluated. In the animal model, propranolol treatment recovered visual dysfunction induced by oxygen exposure, restoring retinal function, as demonstrated by electroretinogram recordings (21). In humans, a recent clinical study showed a poorer refractive status at 1 year of age, in infants previously treated with propranolol (30).

The aim of the present study was to investigate whether propranolol administration in preterm infants with ROP, regardless of its ability to counteract the progression of the ROP, affects refractive status at 1 year of age in former preterm infants.

## Methods

This is a retrospective observational cohort study that included patients with ROP admitted to the Neonatal Intensive Care Unit (NICU) of the Meyer University Children's Hospital in Florence, and Fondazione IRCCS Ca' Granda Ospedale Maggiore Policlinico in Milan, Italy. The study assessed the refractive status of former premature infants with previous ROP treated with propranolol (oral or topic) vs. infants with comparable stages of ROP who had not been treated with propranolol. We evaluated physical development parameters and refractive status at 1 year of corrected age. The study was approved by the institutional ethics committee, and informed consent was obtained from both the parents of all patients.

Newborns admitted to the two NICUs in the period 2010–2017, and treated with propranolol were eligible, but only infants with anthropometrical and ophthalmologic follow-up at 1 year of corrected age (standard of care for all infants with ROP) were included. We matched cases, those who had received oral or topic propranolol treatment, with an equal number of patients admitted in the same period who did not receive any propranolol therapy and represent the control group, with comparable characteristics in terms of stages of ROP, birth weight and gestational age. Newborns treated with oral propranolol were treated with 1 or 2 mg/Kg/day and were enrolled with ROP stage 2 (22). While newborns treated with propranolol eye micro-drops at 0.1% were enrolled with ROP stage 2 (28), those treated with eye micro-drops at 0.2% were enrolled with ROP stage 1 (29). Patients who developed a severe grade of ROP (stage 2 plus or 3 plus) were treated with laser or anti-VEGF antibodies according to the ophthalmologist, regardless of any prior treatment with propranolol. An experienced ophthalmologist specialized in ROP and blinded to the intervention evaluated the patients during hospitalization and then during the follow-up. They recorded the progression and severity of the disease according to International classification (31). The anthropometric (weight, height, head circumference) and ophthalmologic follow-up was performed at 1 year of corrected age. Refractive status was evaluated through a cycloplegic retinoscopy with 1% tropicamide. Spherical equivalents (SE) were determined through skiascopy or handheld autorefractor (Retinomax 3, Nikon Inc., Japan) and measured in diopters (D). Myopia, defined as a SE <0 D, was distinct in mild myopia (0 to −1.5 D), moderate myopia (−1.5 to −6D), and high myopia (> −6 D) (32). Hypermetropia was considered significant if < +3 D.

### Statistical Analysis

To estimate the sample size, we used a web calculator (Sealed Envelope Ltd. 2012). Assuming an α error of 5%, a power value of 80%, a standard deviation of outcome of 1.09 (33) and a non-inferiority limit of 0.6, 82 patients had to be enrolled.

The data were analyzed by using the SPSS 25.0 (SPSS Inc. Chicago, Illinois) statistical package program. Continuous variables are presented as means ± standard deviations and 95% confidence intervals were determined. Nominal variables were presented as numbers and percentages. Comparisons between the approaches were performed with Student's *t*-test and one-way ANOVA for continuous variables and Chi-squared or Fisher's exact test for categorical data. The groups with significant differences compared to continuous variables were *post-hoc* analyzed. The *post-hoc* analysis was performed using one-way ANOVA test with least significant difference (LSD) and Bonferroni methods. A *p* < 0.05 was accepted as statistically significant. The difference in incidence of refractive errors between treated or control groups was evaluated by means of the odds ratio (OR).

## Results

**Figure 1:**
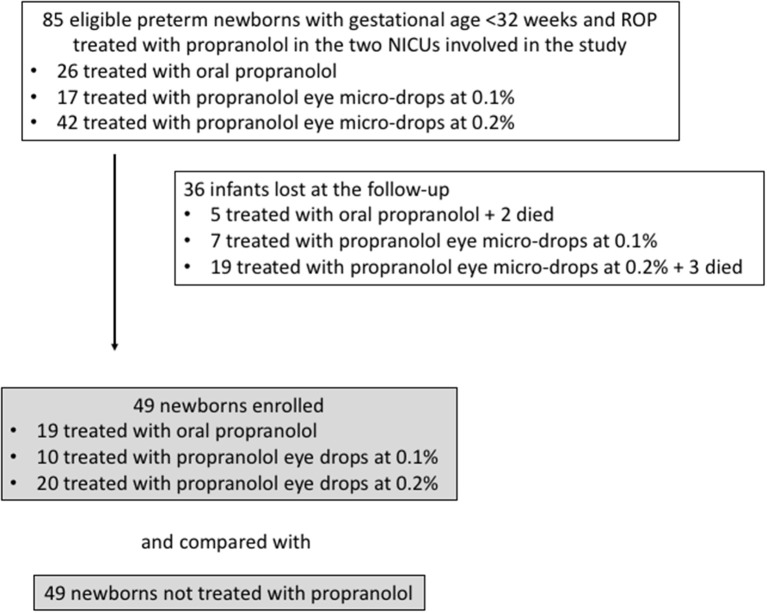
Flow chart illustrating patient enrollment of this retrospective observational cohort study.

Among the 85 eligible newborns (the newborns enrolled in the different clinical trials in the two NICUs involved in the study), 36 were excluded, 5 because they died, and 31 because they were lost at follow-up (Figure 1). The infants lost to follow-up showed baseline data comparable to those of newborns monitored at follow-up (GA 191.5 ± 15.0 days, Birth weight 811 ± 260 g, 10 with stage 1 ROP, 13 with stage 2, 9 with stage 3, and 4 with stage 2 or 3 plus).

The 49 patients enrolled who received propranolol therapy (Treated Group, TG) were matched with 49 patients with equivalent anthropometrical parameters and comparable stages of disease who did not receive any pharmacological treatment and representing the Control Group (CG). Among all newborns of the TG, 19 patients were treated with oral propranolol (4 with 0.25 mg/kg/6 h and 15 with 0.5 mg/kg/6 h, for a mean of 66 ± 30 days), 10 were treated with eye micro-drops at 0.1% (three micro-drops every 8 h in both eyes, for a mean of 60 ± 17 days), and 20 patients received eye micro-drops at 0.2% (three micro-drops every 6 h in both eyes, for a mean of 70 ± 27 days).

Baseline characteristics and anthropometric measurements are described in Table 1. The TG and the CG showed comparable gestational age, weight, length, and head circumference at birth. We found no significant differences in disease progression measured in terms of maximum stages of ROP reached (Table 1). The number of patients who required more invasive treatment (laser phototherapy or anti-VEGF administration) was comparable between the groups, without any significant difference even though the trend toward a reduced progression in TG was evident.

**Table 1:** Baseline characteristics and anthropometric measurements at birth.

	**Treated group**	**Control group**	***p***
Infants, *n*	49	49	
GA, *days*, mean ± DS	184.8 ± 16.0	189.0 ± 18.4	0.228
Birth weight, grams, mean ± DS	804 ± 197	891 ± 288	0.083
Birth head circumference, cm, mean ± DS	23.5 ± 2.6	24.4 ± 2.6	0.091
Birth length, cm, mean ± DS	33.8 ± 3.7	34.8 ± 2.7	0.121
ROP Stage 1, *n* (%)	10 (20.4%)	18 (36.7%)	0.075
ROP Stage 2, *n* (%)	20 (40.8%)	15 (30.6%)	0.297
ROP Stage 3, *n* (%)	12 (24.5%)	5 (10.2%)	0.063
ROP Stage 2 or 3 plus, *n* (%)	7 (14.3%)	11 (22.4%)	0.302
Treated with invasive therapy (anti VEGF or laser), *n* (%)	7 (14.3%)	11 (22.4%)	0.302

**Figure 2:**
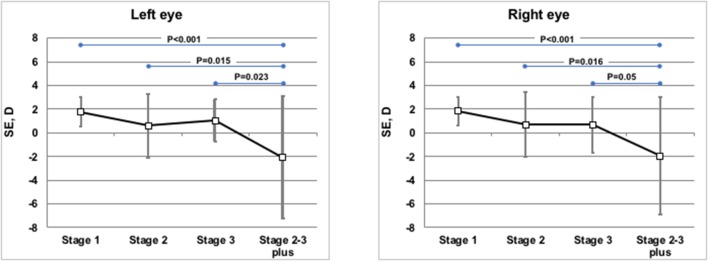
Mean spherical equivalents at 1 year of post-menstrual age in Left or Right Eye in correlation with ROP stages. Vertical lines indicate 95% confidence intervals.

Weight, length, head circumference, and refractive status were measured at similar corrected ages (10.6 ± 2.1 months of post-menstrual age in the TG, 11.3 ± 1.7 months of post-menstrual age in the CG; *p* = 0.069). The anthropometric parameters (weight, length, and head circumference) at 1 year of corrected age were similar between the groups, without statistically significant differences (Table 2). Refractive evaluation at 1 year showed SE values decreasing with ROP progression. In infants with prior ROP stage 2–3 plus who had undergone invasive treatment, the value of SE was significantly lower if compared with infants with prior stage 1, 2, or 3 ROP (Figure 2). The poorer refractive outcome of infants with previous ROP stage 2–3 plus who had undergone invasive treatment is confirmed by the higher number of severe myopia observed in this group (Table 3). Infants with prior stage 2–3 plus ROP show a risk to develop a severe myopia significantly higher than newborns with ROP stages 1–3 (OR 30.385; 95% CI 3.281–281.380; *p* = 0.0026).

**Table 2:** Anthropometric measurements at 1 year of post-menstrual age.

	**Treated group**	**Control group**	***p***
Infants, *n*	49	49	
Weight at 1 year, grams, mean ± DS	7,820 ± 1,100	7,855 ± 1,035	0.871
Length at 1 year, cm, mean ± DS	70.5 ± 4.9	71.1 ± 5.0	0.542
Head circumference at 1 year, cm, mean ± DS	44.0 ± 2.0	43.8 ± 1.3	0.628

**Table 3:** Number of patients with refractive errors correlated with the stages of ROP.

	***n***	**Mild myopia**	**Moderate myopia**	**High myopia**	**Hypermetropia**
Stage 1 ROP	28	2 (7.1%)	0 (0)	0 (0)	4 (14.3%)
Stage 2 ROP	35	7 (20.0%)	7 (20.0%)	1 (2.8%)	4 (11.4%)
Stage 3 ROP	17	3 (17.6%)	2 (11.8%)	0 (0)	2 (11.8%)
Stage 2–3 plus ROP	18	4 (22.2%)	3 (16.7%)	5 (27.8%)	3 (16.7%)

**Figure 3:**
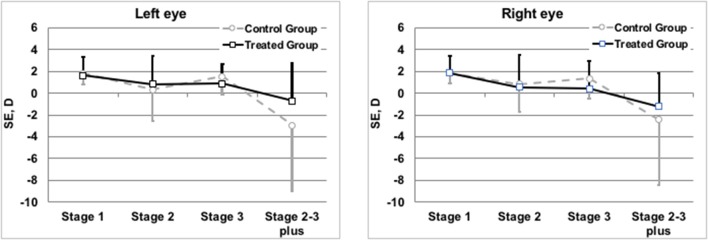
Mean spherical equivalents at 1 year of post-menstrual age in Left or Right Eye in correlation with treatment or not with propranolol. Vertical lines indicate 95% confidence intervals.

Refractive evaluation at 1 year showed a SE in the infants of TG similar to those observed in the CG. Also comparing the SE measured in infants (treated or not with propranolol) at the same stage of severity of the disease, no differences in the refractive outcome were observed (Figure 3). The similar refractive outcome of infants treated, or not, with propranolol is demonstrated by the similar distribution of refractive errors in these two groups (Table 4).

**Table 4:** Number of patients with refractive errors correlated with the treatment or not with propranolol.

	**Treated group**	**Control group**	**OR (95% CI)**	***p***
Infants, *n*	49	49		
Mild myopia, *n*	10 (20.4%)	6 (12.2%)	1.838 (0.611–5.526)	0.279
Moderate myopia, *n*	7 (14.3%)	5 (10.2%)	1.467 (0.432–4.983)	0.614
High myopia, *n*	2 (4.1%)	4 (8.2%)	0.479 (0.083–2.744)	0.408
Hypermetropia, *n*	7 (14.3%)	6 (12.2%)	1.194 (0.371–3.850)	0.766

No statistically significant differences of SE were observed among infants without any pharmacological treatment and infants treated with oral propranolol, or eye micro-drops at 0.1, or at 0.2% (Table 5).

**Table 5:** Refractive evaluation at 1 year of post-menstrual age in newborns with different treatments.

	**Control group**	**Oral propranolol**	**Propranolol eye micro-drops 0.1%**	**Propranolol eye micro-drops 0.2%**	***p***
Infants, *n*	49	19	10	20	
SE LE, *D* (mean ± DS)	+0.25 ± 3.74	+0.47 ± 3.35	+1.08 ± 1.51	+0.93 ± 1.78	NS
SE RE, *D* (mean ± DS)	+0.53 ± 3.57	+0.47 ± 3.23	+0.98 ± 1.42	+0.37 ± 2.71	NS

*SE, Spheric equivalent; LE, Left Eye; RE, Right Eye*.

## Discussion

The possible efficacy of propranolol (oral or topical) in reducing the progression of ROP, on the one hand opens up high hopes for an effective and economical treatment, but on the other hand raises questions about the short- and long-term effects of propranolol treatment. This study reports the anthropometric and refractive outcome at 1 year of post-menstrual age in a series of infants with different stages of ROP, treated or not with propranolol, to obtain information about the possible effect of propranolol in the short-term refractive outcome.

Regarding physical growth, that was comparable between both groups at birth, no difference of anthropometric parameters was observed at 1 year of age between the group that received propranolol and the CG, confirming the data already reported for infants treated with propranolol for infantile hemangiomas (34).

Results of the refractory outcome show values of the SE in line with data of the literature (35) and suggest that, at the same stage of ROP, propranolol itself doesn't affect the refractive outcome. In fact, the number of infants with refractive errors were similar in both the TG and the CG. Results shown in Table 5 indicate that among infants treated with propranolol, mild myopia prevail compared to infants in the control group, while severe myopia seem to be less frequent. However, these data do not reach statistical significance and therefore deserve to be confirmed by more extensive studies. On the contrary, the refractive outcome worsens with the progression of the severity of the ROP, as demonstrated by the high percentage of severe myopia in infants who had undergone invasive procedures, demonstrating that the progression of ROP affects the refractive outcome. This observation is based on a small number of infants, but results are in agreement with previous literature findings (36, 37).

These observations suggest that while propranolol itself does not affect the refractive outcome, the progression of ROP worsens the refractive defects. If these data are confirmed by further study, and if the efficacy of propranolol in counteracting ROP progression is confirmed by further clinical trials, the conclusion will be that propranolol might indirectly improve the visual outcome simply by reducing the progression of ROP.

The results of this study are in apparent contrast with the study of Korkmaz et al., where the authors reported a poorer refractive outcome in newborns treated with propranolol (27–30). It is difficult to provide a specific explanation for this discrepancy. In all the studies, the number of the enrolled infants was small, the route and the dosage of propranolol administration was not homogeneous, and therefore only theoretical speculations are possible. However, the trial performed by Korkmaz et al. differed from those performed in our centers, mainly regarding the moment in which propranolol treatment was started. While all the patients enrolled in our studies were treated with propranolol during the proliferative phase of ROP (during stage 1 or 2 ROP), in the study of Korkmaz, newborns were treated on the basis of their gestational and post-conceptional age. Newborns born before 27 weeks of gestational age started oral propranolol at 32 post-menstrual weeks, those born at 27–29 weeks started propranolol at 33 post-menstrual weeks, and, finally, newborns born at 30 or later gestational weeks, received propranolol at 34 post-menstrual weeks. The consequence of this approach was that, in the study of Korkmaz, 30 newborns were treated with propranolol before ROP appearance, during the ischemic phase, and 53 newborns during the proliferative phase (24, 25). The study that explored the visual outcome of these newborns analyzed 34 of the 83 newborns treated with propranolol, but unfortunately, in this article, it was not reported how many of these patients started treatment during the proliferative phase or the ischemic phase.

Recently, we have demonstrated that the phase in which propranolol is started affects its effectiveness. In fact, propranolol administered during the proliferative phase has a protective effect, with an evident reduction in ROP progression, while the administration of propranolol during the ischemic phase exerts a probable deleterious effect (26–29). This apparently contradictory effect is definitely explainable by the bi-phasic and specular nature of ROP. It is, in fact, easy to understand that the administration of propranolol, a molecule able to reduce the production of VEGF, may be useful during the proliferative phase of ROP, when VEGF is dramatically increased, and a reduction of VEGF is desirable, but detrimental if administered during the ischemic phase, when levels of VEGF are too low to permit a normal vascularization of the retina, and an increase of VEGF is suitable. In light of these results, we speculated that propranolol is only useful during the proliferative phase of ROP (26–29). In conclusion, we cannot exclude that the discrepancy of the results between these two studies may be related to the different strategy adopted.

This study has some evident limitations which have to be pointed out. The most important flaws of this study are the retrospective design, the small number of patients analyzed, and the low follow-up rate (58%) of propranolol-treated infants, due to the fact that many infants were followed in centers closer to their cities of residence. The populations compared were not perfectly homogeneous: in the CG the number of infants who developed stage 2–3 plus was greater than in the TG. Even if this difference has not reached the statistical significance, we cannot exclude that this lack of homogeneity has conditioned the results of the refractive outcome. Moreover, although all the patients treated with propranolol received this treatment during the proliferative phase, the route of administration and the dose were markedly different, with different plasma concentration. Finally, the visual follow-up was restricted to a first year of post-menstrual age. Therefore, further studies, possibly prospective placebo-controlled trials, with a larger sample size are urgently warranted.

## Conclusion

In conclusion, this study suggests that propranolol administered during the proliferative phase of ROP does not affect the refractive outcome, while the progression of ROP does. If further studies confirm that propranolol can counteract ROP progression, it will be possible to conclude that propranolol indirectly improves the visual outcome.

## Data Availability Statement

The raw data supporting the conclusions of this manuscript will be made available by the authors, without undue reservation, to any qualified researcher.

## Ethics Statement

The studies involving human participants were reviewed and approved by Ethical Committee of Meyer University Children's Hospital, Florence and of Fondazione IRCCS Ca' Granda Ospedale Maggiore Policlinico, Milan, Italy. Written informed consent to participate in this study was provided by the participants' legal guardian/next of kin.

## Author Contributions

LF and GC conceptualized and designed the study, drafted the initial manuscript, and reviewed and revised the manuscript. LP, ES, GA, GRe, and GRa, contributed to patients' enrolment, neonatal monitoring, and acquisition of data. PB and MD contributed to conception and design of the study, contributed to the drafting the manuscript. MC and SD contributed to the analysis of the data and had primary responsibility for statistical analysis. PF and SO contributed to patients' enrolment, contributed to ophthalmologic evaluations, and acquisition of data. FM contributed to conception and design of the study, supervised the design and the execution of the study, and critically reviewed the manuscript for important intellectual content. All authors approved the final manuscript as submitted and agree to be accountable for all aspects of the work.

### Conflict of Interest

The authors declare that the research was conducted in the absence of any commercial or financial relationships that could be construed as a potential conflict of interest.

## Abbreviations

ROP: retinopathy of prematurity
VEGF: vascular endothelial growth factor
IGF-1: insulin growth factor-1
β-AR: β-adrenoreceptor
NICU: neonatal intensive care unit
SE: spherical equivalents
D: diopters
TG: treated group
CG: control group.
